# Examining the impact of perceived stress, anxiety, and resilience on depression among medical staff after COVID-19 quarantine: a chain mediation analysis

**DOI:** 10.3389/fpubh.2023.1250623

**Published:** 2023-09-19

**Authors:** Dongyang Chen, Yi Ni, Jiani Lu, Yiwen Wang, Qi Qi, Hua Zhai

**Affiliations:** Shanghai YangZhi Rehabilitation Hospital (Shanghai Sunshine Rehabilitation Center), School of Medicine, Tongji University, Shanghai, China

**Keywords:** COVID-19, perceived stress, anxiety, resilience, depression

## Abstract

**Introduction:**

The COVID-19 pandemic and subsequent quarantine measures have led to a significant impact on mental health worldwide. Medical staff, in particular, have been exposed to high levels of stress due to their frontline work during the crisis. However, there is still limited research on the psychological mechanism among medical staff after quarantine.

**Methods:**

In this cross-sectional observational study, 150 medical staff from Shanghai YangZhi Rehabilitation Hospital, Shanghai, China, were enrolled in October 2022. SPSS 26.0 and PROCESS 4.0 model 6 were used to analyze the chain mediating effect of perceived stress, anxiety, resilience and depression among medical staff after quarantine. Anxiety and depression were compared during and after the quarantine. All scales have high validity and reliability in a Chinese population.

**Results:**

Our findings revealed a positive correlation between perceived stress and anxiety (r = 0.60, *p* < 0.001) and depression (*r* = 0.60, *p* < 0.001) levels among medical staff. Conversely, resilience was found to have a negative correlation with perceived stress (*r* = −0.67, *p* < 0.001), anxiety (*r* = −0.57, *p* < 0.001) and depression (*r* = −0.61, *p* < 0.001). The score of depression during the quarantine was higher than the score after the quarantine, but the *p*-value is only marginally significant (*p* = 0.067). The score of anxiety during the quarantine was significantly higher than the score after the quarantine (*p* < 0.05). Moreover, the chain mediation model suggested that anxiety and resilience could mediate the association between perceived stress and depression among medical staff following quarantine. Specifically, perceived stress had no direct effect on depression (β = 0.025, *t* = 0.548, *p* = 0.59) but positively predicted anxiety (β = 0.381, *t* = 8.817, *p* < 0.001) and resilience (β = −1.302, *t* = −6.781, *p* < 0.001), which influenced depression levels indirectly through multiple pathways. The three indirect paths: the mediating role of anxiety, the mediating role of resilience, and the chain mediating role of both anxiety and resilience.

**Discussion:**

This study emphasizes the importance of psychological interventions aimed at protecting medical staff’s psychological resilience and promoting coping mechanisms to manage stress during and after crises such as the COVID-19 pandemic. Additionally, our findings suggest that both anxiety and resilience play critical roles in mitigating the detrimental effects of perceived stress on mental health and further highlight the need for continued research to better understand the complex interplay of these factors.

## 1. Introduction

The impact of the COVID-19 pandemic on people’s mental health is far-reaching, and healthcare workers are no exception. To address the challenges posed by the outbreak, hospitals in affected regions of China have implemented extensive measures such as lockdowns and closures. For most people, quarantine is a distasteful experience associated with a range of problems, including fears of infection and inadequate financial supplies (1–3). Previous studies have shown that quarantine can dramatically affect individuals’ mental health (4), and may even lead to suicide (5). However, there is still relatively little research on people’s mental health states following the lifting of quarantine measures.

Healthcare workers have been exposed to an increase in highly stressful clinical situations due to COVID-19 (6), leading to chronic stress (7). Numerous studies have highlighted the impact of occupational stress on healthcare workers’ mental health during the pandemic (6, 8, 9). While a strong relationship between stress and depression is well-known, the psychological mechanisms underlying this relationship remain unclear. For example, although stress can increase the risk of depression, not everyone experiences depression in the face of pressure. This key issue has drawn considerable attention but remains unresolved.

There are many risk factors in working conditions impacting mental health. According the Resource-Coping Model, work stress can deplete an individual’s resources, leading to a decrease in coping ability, and thus affecting mental health (10). Resources can be personal traits, social support, emotions, etc. Female healthcare workers are more vulnerable to mental health problems (e.g., depression) (11). Moreover, fatigue was also a significant predictor of depression (12). Research has extensively explored methods to mitigate the negative impact of work stress on mental health. Systematic reviews have highlighted the protective role of adaptive coping strategies for individuals experiencing burnout due to long-term work stress (13, 14). Additionally, there were another research found that off-job crafting can act as a buffer mechanism against burnout during the COVID-19 crisis (15). In fact, these methods are more or less related to psychological resilience. Patrizia et al. found that higher levels of resilience were associated with a greater utilization of adaptive coping behaviors and a decreased reliance on maladaptive coping behaviors (16).

Increasing studies suggest a strong relationship between higher levels of psychological resilience and lower levels of depression (17, 18). Although there is not a universally accepted definition of psychological resilience, it’s known as the ability to cope with adversity and daily stress, which varies significantly among individuals and greatly depends on environmental and personal factors (19). Some scholars have emphasized the mediating role of psychological resilience in the relationship between stress and depression (20). The prevalence of depression and anxiety has increased after COVID-19 (21), making the protection of psychological resilience critical (17). Scholars have confirmed that resilience is a significant predictor of perceived stress (22). Researchers have found that quarantine can greatly predict acute stress disorder and high levels of depression symptoms in medical staff, even after long-term quarantine (23, 24). Nevertheless, there is limited research on how psychological resilience affects the relationship between stress and depression in the medical staff after quarantine.

Previous studies have shown that the psychological resilience acts a mediator. As such, it is feasible to assume that resilience may mediate the relationship between stress and depression among medical staff following quarantine. Anxiety and depression are often linked as closely related symptoms. Around 85% of people with depression also experience anxiety, while depression occurs in up to 90% of patients with anxiety disorders (25). In fact, work stress was often associated with anxiety and depression symptoms in both men and women (26). It seems that depression and anxiety do not develop simultaneously after perceiving stress. According to the Cognitive Activation Theory of Stress (CATS) (27), individuals may feel discomfort and negative emotions, such as anxiety, when confronted with stress. However, through coping and self-regulation, they can restore their emotional state to normal levels. On the other hand, if an individual remains in a state of high tension and unable to find solutions over a prolonged period, this may lead to negative emotions such as depression. Studying whether anxiety and psychological resilience, as an emotional state and internal driving force, respectively, can impact the relationship between perceived stress and depression among medical staff is worthwhile.

**Figure 1:**
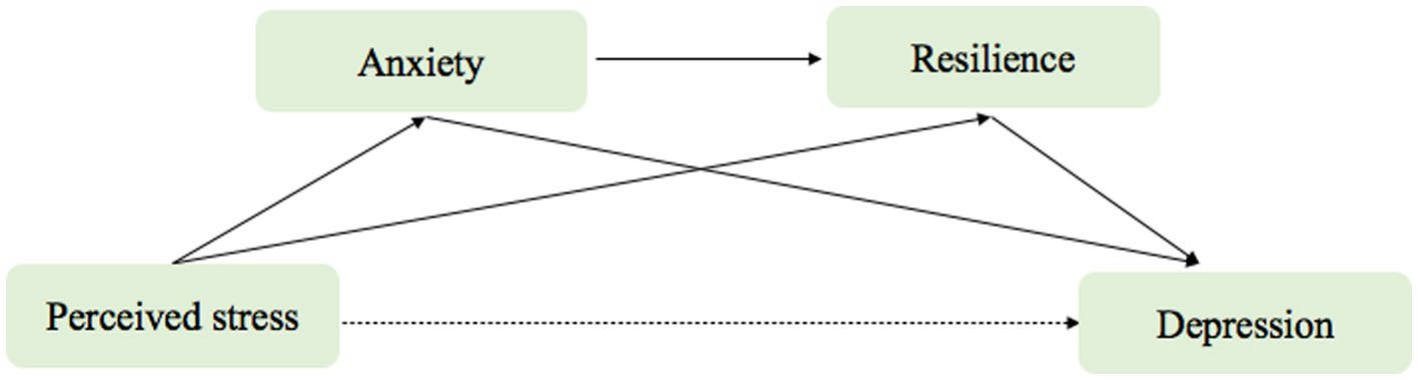
Hypothesized relationships between perceived stress, anxiety, resilience and depression.

The purpose of this study was to examine perceived stress, anxiety, psychological resilience, and depression among medical staff in China after quarantine. We also aimed to compare depression and anxiety levels before and after quarantine closure. Furthermore, we investigated a chain mediating model, whereby anxiety and psychological resilience mediate the relationship between perceived stress and depression (Figure 1).

## 2. Methods

### 2.1. Design and participants

This study is a cross-sectional study. The sample method used was convenience sampling. All samples were from medical personnel who had participated in bubble type closed management during the COVID-19. This management approach involved full closure, point to point management and complete monitoring throughout the entire process. The survey was in the form of electronic questionnaires and was distributed to medical personnel via social media platform (WeChat, Tencent). The template of the electronic questionnaire was provided by the application “Questionnaire Star” that collected valid questionnaire results. All subjects participated voluntarily and all data was collected anonymously.

Based on the previous study, the prevalence of depression symptoms was 18.4% among Chinese medical staff (28). The allowable error is set at 5%, with a confidence level of 1-α = 0.80. It is calculated that a sample size of 117 is needed for the survey. Assuming a non-response rate of 10% and a questionnaire pass rate of 80%, a sample size of 147 is required. The sample size computation was carried out suing PASS 21 (29). Inclusion criteria were as follows: (1) all participants were the medical staff; (2) all participants were willing to cooperate with the study. Exclusion criteria were as follows: (1) mental illness; (2) cognitive dysfunction.

### 2.2. Ethical considerations

Online informed consent was obtained from all participants on the information page before filling out the questionnaire. Participants were also informed of their right to withdraw from the survey at any time. The collected data was safely stored in the specific computer server with password protection in the Shanghai YangZhi Rehabilitation Hospital, Shanghai, China. The survey was approved by the Ethics Committee of Shanghai YangZhi Rehabilitation Hospital (Shanghai Sunshine Rehabilitation Center), School of Medicine, Tongji University (No. 2022–027, October 2022).

### 2.3. Variables and measurements

The questionnaire consisted of four parts. The first part focused on demographics, such as gender (0 = male, 1 = female), age (years), education level (0 = specialized subject, 1 = undergraduate, 2 = postgraduate), professional title (0 = none, 1 = junior, 2 = intermediate, 3 = depute senior or above), length of work (years), monthly household income (0 = less than 10,000 yuan, 1 = 10,000 to 20,000 yuan, 2 = 20,000 to 30,000 yuan, 3 = more than 30,000 yuan), marital status (0 = single, 1 = married), number of children (0 = not a one, 1 = one, 2 = two or more), occupation type (0 = doctor, 1 = nurse, 2 = therapist), and number of hobbies (0 = not a one, 1 = one, 2 = two or more). The development of items is based on previous literature and clinical experience, and then discussed by several members of the research team.

The second part was the Patient Health Questionnaire (PHQ-9) and the General Anxiety Disorder Scale (GAD-7) scales, which was used to evaluate the current (after the quarantine) and previous (during the quarantine) levels of depression and anxiety experienced by medical staff. The third part utilized the Perceived Stress Scale-14 (PSS-14) and the Connor-Davidson Resilience Scale (CD-RISC) scales to measure the current and previous levels of stress and psychological resilience among medical staff. All scales have a Chinese version, which is adapted to the Chinese population.

#### 2.3.1. The patient health questionnaire

The Patient Health Questionnaire, which covers the DSM-5 diagnostic criteria for depressive disorder, can both assess the severity of depression and have potential diagnostic efficacy (30). The PHQ-9 scale has 9 items on a 3-point Likert scale, with a total score of 27 points. The cutoff values are 5, 10, 15, and 20 points, corresponding to mild, moderate, moderately severe, and severe levels, respectively. The Chinese version of the PHQ-9 shows good reliability and validity (31) with a Cronbach’s alpha of 0.920.

#### 2.3.2. The general anxiety disorder scale

The Generalized Anxiety Disorder Scale was used to assess the severity of anxiety symptoms (32). The GAD-7 scale has 7 items on a 3-point Likert scale, with a total score of 21 points. The thresholds for mild, moderate, and severe anxiety are 5, 10, and 15 points, respectively. In this study, we used the Chinese version of GAD-7 translated and revised by Xiaoyan et al. The Chinese version of GAD-7 shows good reliability and validity (33) with a Cronbach’s alpha of 0.926.

#### 2.3.3. The Perceived stress scale

The Perceived Stress Scale was used to assess perceived stress (34). The scale measured two dimensions: loss and tension, quantifying the extent of self-awareness of stress and belief that one’s life had been overloaded or unpredictable or uncontrollable during the prior month. Respondents answered 14 items on a 5-point Likert scale, with a higher score indicating greater mental stress. The minimum and maximum points were 14 and 70, respectively. The Chinese version of the PSS has shown high validity and reliability (35) with a Cronbach’s alpha of 0.673. The Cronbach’s Alpha coefficient for the sub-dimension “tension” is 0.766, and for the sub-dimension “loss” is 0.890.

#### 2.3.4. The Connor-Davidson Resilience Scale

Psychological resilience was assessed with the Connor-Davidson Resilience Scale, which measures three factors: toughness, strength, and optimism (36). The scale consists of 25 items rated on a 5-point Likert scale ranging from 1 (not at all) to 4 (always). A higher score, with a possible range of 0 to 100 points, indicates greater resilience. The scale has shown high validity and reliability in a Chinese population (37) with a Cronbach’s alpha of 0.963.

### 2.4. Statistical analysis

We used SPSS 26.0 software package, and the IBM SPSS macro program PROCESS, version 4.0, for statistical analysis in this study. We conducted descriptive statistics on all basic demographic data and performed correlation analysis on all psychological variables. One-sample Kolmogorov Smirnov tests were used to check the normality of distributions for the continuous variables. The group comparisons regarding continuous variables were analyzed by using T-test for normally distributed data or Wilcoxon signed-rank test for skewed data. Pearson correlation or spearman’s rank correlation was used to examine the relationships between all psychological variables. For the analyses of chain mediating effect, we used Model 6 in PROCESS, version 4.0. Finally, bootstrapping with 5,000 resamples and a 95% confidence interval (CI) was used to analyze the significance of the chain mediating model. *p* value less than 0.05 indicated statistical significance.

## 3. Results

### 3.1. Demographic characteristics

A total of 150 medical staff members participated in this study, consisting of 34 men (22.7%) and 116 women (77.3%). The main age group of participants was between 25–40 years old (89.3%). Of the participants, 89 (59.3%) were single and 61(40.7%) were married. There were 16 doctors (10.7%), 73 nurses (48.7%) and 61 therapists (40.7%). There were 100 candidates with undergraduate degrees (66.7%), 43 with postgraduate degrees (28.7%), and 7 with other degrees (4.7%). Sixty had one child (40%), 16 had two or more children (10.7%), and 74 had none of them (49.3%). For more specific demographic characteristics, see Table 1.

**Table 1:** Sociodemographic characteristics of participants.

Variables	*n*	%
*Gender*		
Male	34	22.7
Female	116	77.3
*Age*		
<25	7	4.7
25–40	134	89.3
>40	9	6.0
*Marital status*		
Single	89	59.3
Married	61	40.7
*Number of children*		
Not a one	74	49.3
One	60	40.0
Two or more	16	10.7
*Monthly household income*		
Less than 10,000 yuan	15	10.0
10,000 to 20,000 yuan	74	49.3
20,000 to 30,000 yuan	42	28.0
More than 30,000 yuan	19	12.7
*Number of hobbies*		
Not a one	15	10.0
One	108	72.0
Two or more	27	18.0
*Educational background*		
Specialized subject	7	4.7
Undergraduate	100	66.7
Postgraduate	43	28.7
*Occupation type*		
Doctor	16	10.7
Nurse	73	48.7
Therapist	61	40.7
*Title*		
None	6	4.0
Junior	88	58.7
Intermediate	50	33.3
Deputy senior or above	6	4.0
*Length of work*		
≤1	16	10.7
2–4	27	18.0
5–9	43	28.7
10–19	56	37.3
≥20	8	5.3

### 3.2. The correlation and comparison

**Figure 2:**
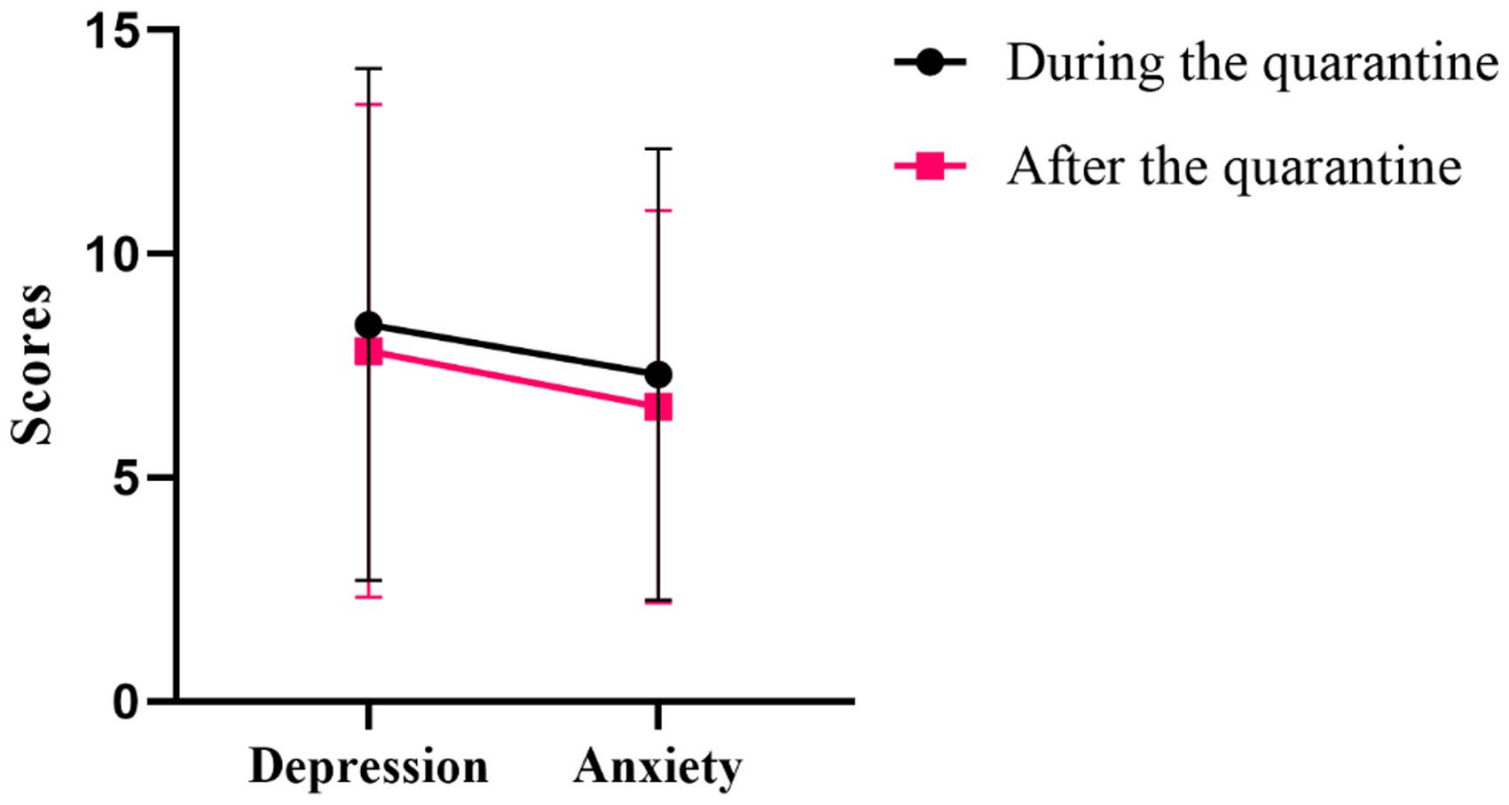
The comparison of levels of depression and anxiety during quarantine and after quarantine among medical staff.

Table 2 presents the results of the correlational analysis of perceived stress, psychological resilience, depression and anxiety of participants. Participants scored an average of 25.40 ± 6.85 on the PSS, 85.80 ± 17.07 on the CD-RISC, 6.58 ± 4.38 on the GAD-7, 7.84 ± 5.51 on the PHQ-9. All psychological variables are skewed data by using the One-sample Kolmogorov Smirnov tests. It was found that perceived stress was negatively correlated with psychological resilience (*r* = −0.68, *p* < 0.001), whereas perceived stress was positively correlated with depression (*r* = 0.64, *p* < 0.001) and anxiety (*r* = 0.64, *p* < 0.001). Furthermore, psychological resilience was negatively correlated with depression (*r* = −0.66, *p* < 0.001) and anxiety (*r* = −0.61, *p* < 0.001). Figure 2 presents the results of the comparison with the Wilcoxon signed-rank test of depression and anxiety levels among participants during and after quarantine. The score of depression during the quarantine was higher than the score after the quarantine, but the *p*-value is only marginally significant (*p* = 0.067). The score of anxiety during the quarantine was significantly higher than the score after the quarantine (*p* < 0.05).

**Table 2:** Descriptive analysis and correlations.

	1	1.1	1.2	2	2.1	2.2	2.3	3	4
1. Perceived stress	1								
1.1. Tension	0.73***	1							
1.2. Loss	0.79***	0.22***	1						
2. Resilience	−0.68***	−0.25***	−0.76***	1					
2.1. Tenacious	−0.67***	−0.27***	−0.74***	−0.97***	1				
2.2. Strength	−0.66***	−0.24***	−0.74***	0.95***	0.87***	1			
2.3. Optimistic	−0.50***	−0.14	−0.59***	0.79***	0.68***	0.73***	1		
3. Anxiety	0.64***	0.46***	0.48***	−0.61***	−0.55***	−0.62***	−0.58***	1	
4. Depression	0.64***	0.45***	0.51***	−0.66***	−0.60***	−0.67***	−0.61***	0.82***	1
*M*	25.40	13.89	11.51	85.80	43.31	29.00	13.49	6.58	7.84
*SD*	6.85	4.06	4.61	17.07	9.57	5.69	2.87	4.38	5.51

*p < 0.05, **p < 0.01, ***p < 0.001. Spearman’s rank correlation coefficient analysis method is used to analyze the correlation relationship between all variables.

### 3.3. Chain mediation effects analysis

This study examined the chain mediating role of anxiety and resilience on perceived stress and depression (Table 3). In our study, the direct and indirect effects refer to the different pathways through which variables influence each other. The direct effect represents the relationship between two variables without any intervening variables, while the indirect effect represents the relationship between two variables that is mediated by one or more intermediate variables. To analyze the indirect effects, we used the Bootstrap confidence interval method (38), which is a method for determining the significance of mediation effects. It generates confidence intervals by repeatedly resampling the sample data, thereby estimating the true value of the mediation effect. If the confidence interval does not contain zero, it indicates that the mediation effect is significant. Perceived stress was used as an independent variable, depression was used as a dependent variable, anxiety and resilience was used as mediating variables, and the PROCESS Macro 4.0 model 6 was used to test the chain mediating effect (Table 4).

**Table 3:** Regression analysis of variables’ relationships in the model.

	Anxiety				Resilience				Depression			
	*β*	*SE*	*t*	95%CI	*β*	*SE*	*t*	95%CI	*β*	*SE*	*t*	95%CI
Perceived stress	0.381	0.043	8.817**	0.295,0.466	−1.302	0.192	−6.781**	−1.682, −0.922	0.025	0.046	0.548	−0.066, 0.117
Anxiety					−1.042	0.303	−3.434**	−1.642, −0.442	0.966	0.066	14.686**	0.836, 1.096
Resilience									−0.047	0.018	−2.658**	−0.083, −0.012
*R* ^2^	0.362				0.497				0.783			
*F*	77.735**				67.239**				162.18**			

*p < 0.05, **p < 0.01.

**Table 4:** The analysis of the chain mediating effect of anxiety and resilience to perceived stress and depression.

Pathways	Effect	Boot SE	Percentage of mediating effect (%)	95%CI
Perceived stress → Depression	0.025	0.046		−0.066, 0.117
Perceived stress → Anxiety → Depression	0.368	0.065	82.14	0.261, 0.493
Perceived stress → Resilience → Depression	0.062	0.028	13.83	0.008, 0.119
Perceived stress → Anxiety → Resilience → Depression	0.019	0.011	4.24	0.002, 0.045
Total mediating effect	0.448	0.068		0.324, 0.581
Total effect	0.474	0.055		0.366, 0.582

**Figure 3:**
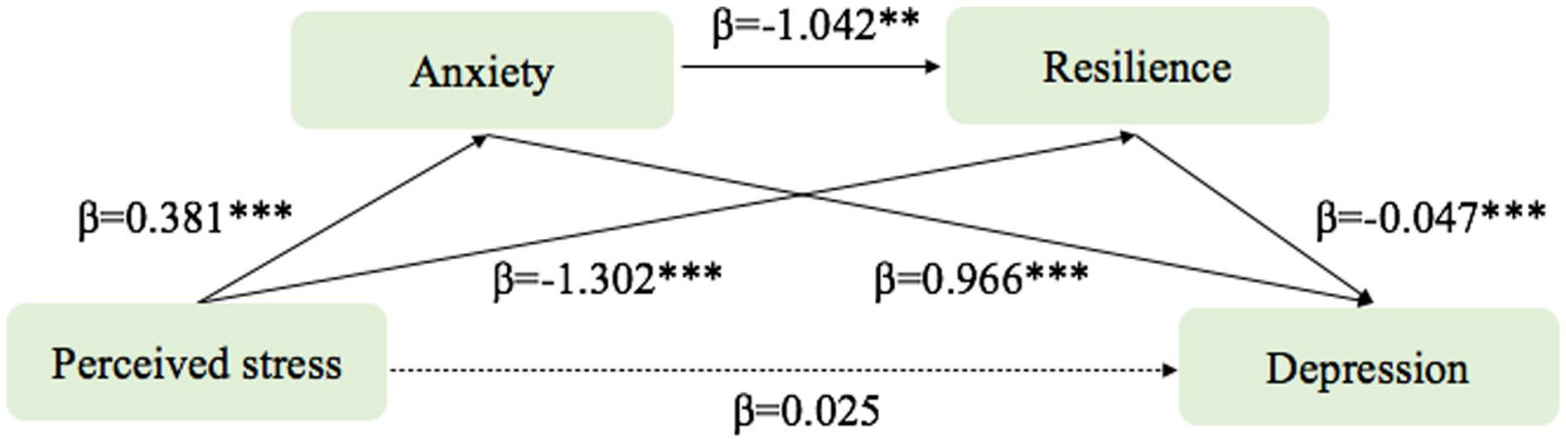
The role of anxiety and resilience as chain mediators in the relationship between perceived stress and depression with standardized beta (IBM SPSS macro program PROCESS v4.0 Model 6). *p < 0.05, **p < 0.01, ***p < 0.001. Values on paths are path coefficients (standardized βs).

The results indicated that:(1) Perceived stress did not have a significant direct predictive effect on depression, with a direct effect value of 0.025 (95% CI [−0.066, 0.017]); (2) Perceived stress had a significant indirect predictive effect on depression *via* anxiety, with an indirect effect value of 0.368 (95%CI[0.261, 0.493]); (3) perceived stress had a significant indirect predictive effect on depression through resilience, with an indirect effect value of 0.062 (95% CI [0.008, 0.119]); (4) perceived stress had a significant indirect predictive effect on depression through anxiety and resilience, with an indirect effect value of 0.019 (95% CI[0.002, 0.045]). A bias-corrected percentile bootstrap method with 5,000 replicate samples was used to determine the role of anxiety and resilience in mediating the chain variables between perceived stress and depression. The standardized path coefficients were calculated to reduce Type 1 errors due to distribution. The results of the chain mediation model analysis are shown in Figure 3.

## 4. Discussion

The present study explored the relationship between the perceived stress, anxiety, psychological resilience and depression among medical staff in China following the quarantine period. Additionally, the study explored the mediating effects of anxiety and psychological resilience on the relationship between perceived stress and depression. The results indicated that perceived stress did not have a direct impact on the depression of medical staff, but indirectly affected depression through anxiety and psychological resilience. Three specific mediation paths were found: the first path is anxiety as a mediation variable, the second is psychological resilience as the mediation variable, and the third is anxiety and psychological resilience as the mediation variables and the chain mediation path. Meanwhile, we investigated that the level of stress, depression and anxiety of medical staff were relatively moderate and low after the quarantine period. Besides, we also asked the participants to recall their levels of depression and anxiety during the quarantine period, and found that they were slightly higher during the lockdown than after it, even though the depression shows marginal significance. Unfortunately, we were unable to investigate the status of perceived stress and psychological resilience during the lockdown period, and therefore cannot compare it with the data after the lockdown. Nonetheless, this still can help improve understanding of the mental health status of medical staff after the pandemic lockdown and inform relevant institutions’ efforts to alleviate and improve the depression phenomenon among healthcare workers.

This study found that anxiety partially mediated the effects of perceived stress and depressive symptoms, which is consistent with previous research on anxiety mediating the effects of stress and self-esteem on depression (39). The results indicated that if medical staff perceive more stress, their emotional state may change and they may feel more anxiety in life, and this anxiety can more produce depressive symptoms. Healthcare workers in China face unique challenges such as high work pressure, frequent interpersonal contact, irregular schedules, and high occupational risk (40, 41). Accumulated perceived stress among healthcare workers can affect job satisfaction, physical health and increase post-traumatic symptoms (42), which may have long-term psychological consequences (43). Previous studies showed that individuals in isolation or quarantine experience high levels of anxiety and stress (4). This study further indicates that medical staff may first experience anxiety symptoms before experiencing depression due to perceived stress.

In addition, this study also found that psychological resilience partially mediated the relationship between perceived stress and depression symptoms among medical staff. This is consistent with previous research on the mediating role of psychological resilience between perceived stress and depression (44). Moreover, psychological resilience was negatively correlated with perceived stress and depression, indicating that medical staff with lower levels of psychological resilience are at higher risk of experiencing stress and depressive symptoms. The mediation analysis suggested that psychological resilience may play a proactive role in buffering the negative effects of perceived stress on depressive symptoms among medical staff (45). These findings contribute to a better understanding of the mental health status of medical staff after the quarantine period.

This study revealed a significant pathway linking perceived stress to anxiety, psychological resilience, and depression. According to the model proposed in this study, the chain relationship between anxiety and psychological resilience mediates the relationship between perceived stress and depression. Numerous studies have demonstrated that perceived stress can lead to higher levels of anxiety (46), and there is a significant negative correlation between anxiety and psychological resilience (20). In other words, medical staff who perceive high levels of stress are more likely to experience prolonged anxiety, which may further impair their psychological resilience and increase their risk of depression. The model suggests that impaired resilience resulting from prolonged stress is likely attributable to the anxiety state it engenders, highlighting the importance of managing stress and anxiety to promote the psychological well-being of medical staff.

Healthcare workers are indispensable members of society, providing critical care to individuals. However, their work is often associated with high levels of stress and anxiety, which can impair their mental health and performance, especially in COVID-19 (47, 48). Therefore, understanding the psychological mechanisms among healthcare workers is vital for maintaining high-quality rehabilitation care. Governments, hospitals, and relevant organizations must recognize this issue and take necessary measures to support frontline workers. For instance, Xu et.al found that Employ Assistance Programs (EAPs) in hospitals can alleviate and reduce the psychological pressure placed on medical staff by actively adopting intervention and guidance measures (49).

This study has some limitation that need to be acknowledged. Firstly, being a cross-sectional study, it only demonstrates the correlation between variables, not causality. Future research should use longitudinal research methods to establish causality. Secondly, all the questionnaires used in this study are self-reported, which may introduce social desirability, response, and recall biases. Thirdly, the study sample was relatively small, with a limited proportion of doctors, which means that the results could have certain limitations. Fourth, a convenience sample was used, which may limit the generalizability of the results. Future research could use a more representative sample to increase the external validity of the findings. Finally, although this study discussed the mediating relationship between variables, it did not analyze the influence of some potential moderating variables, such as attentional control on depression (50). Subsequent studies can conduct in depth analysis of cognitive variables that may affect the two mediators of anxiety and psychological resilience.

## 5. Conclusion

This study is the first to explore the correlation between perceived stress, anxiety, psychological resilience, and depression among medical staff after quarantine. It is clear from the findings that medical staff experience more depressive and anxiety symptoms during the quarantine than after the quarantine. This study further explored the mediating effect of anxiety and psychological resilience on the relationship between perceived stress and depression as well as its chain mediating effect. The results concluded that perceived stress indirectly predicts depression in medical staff through the mediating effect of anxiety, psychological resilience and the chain mediating effect between anxiety and psychological resilience.

## Data availability statement

The raw data supporting the conclusions of this article will be made available by the authors, without undue reservation.

## Ethics statement

The studies involving humans were approved by the Ethics Committee of Shanghai YangZhi Rehabilitation Hospital (Shanghai Sunshine Rehabilitation Center), School of Medicine, Tongji University. The studies were conducted in accordance with the local legislation and institutional requirements. The participants provided their written informed consent to participate in this study.

## Author contributions

Data collection were conducted by DC, YN, JL, and YW. Analysis and interpretation of data were done by DC, YN, JL, YW, QQ, and HZ. Drafting of the paper was performed by DC, YN, QQ, and HZ. Statistical analysis was carried out by DC and YN. Critical revision of the paper was executed by QQ and HZ. All authors contributed to the article and approved the submitted version.

## Conflict of interest

The authors declare that the research was conducted in the absence of any commercial or financial relationships that could be construed as a potential conflict of interest.

## Publisher’s note

All claims expressed in this article are solely those of the authors and do not necessarily represent those of their affiliated organizations, or those of the publisher, the editors and the reviewers. Any product that may be evaluated in this article, or claim that may be made by its manufacturer, is not guaranteed or endorsed by the publisher.
